# Effects of gum chewing exercise on maximum bite force according to facial morphology

**DOI:** 10.1002/cre2.102

**Published:** 2018-02-22

**Authors:** Manami Shirai, Nobuhiko Kawai, Natsuko Hichijo, Masahiko Watanabe, Hiroyo Mori, Silvia Naomi Mitsui, Akihiro Yasue, Eiji Tanaka

**Affiliations:** ^1^ Department of Orthodontics and Dentofacial Orthopedics Institute of Biomedical Sciences, Tokushima University Graduate School Japan

**Keywords:** bite force, craniofacial morphology, gum chewing exercise

## Abstract

Development of the masticatory system is influenced by functional needs. Furthermore, masticatory exercise can improve masticatory function. The aim of this study was to evaluate the potential effect of the gum chewing exercise on the maximum bite force (MBF) in adult subjects with different facial morphologies. MBF was measured by a portable occlusal force gauge and lateral cephalogram was used for evaluation of craniofacial morphology in 19 individuals (7 males and 12 females) with a mean age of 25.4 years (SD ± 4.3). The volunteers underwent gum chewing exercise for 5 min twice a day for 4 weeks. MBF was measured before (T1) and after the 4‐week exercise (T2). The facial morphology of the subjects was classified into the brachy (n = 7), mesio (n = 7), and dolicho (n = 5) facial types. In all three groups, exercise was associated with a significant increase in MBF, though the percent increase was highest in the dolicho facial type. We conclude that gum chewing exercise can improve masticatory performance, especially in individuals with dolicho facial morphology.

## 1 INTRODUCTION

Facial morphology is determined by both genetic and local environmental factors. In addition, mastication also has a substantial effect on craniofacial morphology. Previous studies pointed to a correlation between the maximum bite force (MBF) and facial morphology (Abu Alhaija, Al Zo'ubi, Al Rousan, & Hammad, 2010; Braun et al., 1995; Proffit, Fields, & Nixon, 1983; Ringqvist, 1973; Sondang et al., 2003; Szymańska & Sidorowicz, in press). The mean bite force was twice as great in the normal as in long face subjects (Proffit et al., 1983). Sondang et al. (2003) reported that a larger bite force implies a greater mandibular plane angle and smaller gonial angle, suggesting that long face subjects exert a lower level of bite force. Thus, subjects with dolicho facial morphology are likely to have inferior masticatory efficiency (Gomes, Custodio, Jufer, Del Bel, & Garcia, 2010).

The masticatory system develops, at least in part, in response to functional needs (Kawai et al., 2010). Maki, Nishioka, Morimoto, Naito, and Kimura (2001) reported that a change in diet habit can result in a decrease in bite force in school‐age children. Therefore, masticatory exercise seems important in improving masticatory disturbance and deficiency. It has been reported that gum chewing exercise is helpful in enhancing bite force and masticatory function (Kiliaridis, Tzakis, & Carlsson, 1995; Masumoto, Yamaguchi, & Fujimoto, 2009; Nakagawa et al., 2017; Ohira, Ono, Yano, & Takagi, 2012; van Bruggen et al., 2015). Kiliaridis et al. (1995) reported that 4‐week training with hard chewing gum influenced the functional capacity of the masticatory muscles and increased their strength. Moreover, gum chewing exercise for 4 weeks in Duchenne muscular dystrophy patients improved their masticatory performance (van Bruggen et al., 2015). To our knowledge, however, the effectiveness of the gum chewing exercise on MBF based on craniofacial morphology has never been examined in the past. The aim of this study was to evaluate the effect of chewing exercise on MBF of adult subjects with different facial morphologies.

## 2 METHODS

### 2.1 Subjects

Nineteen individuals, dental school students and staff members, at Tokushima University (7 males and 12 females) with a mean age of 25.4 ± 4.3 years (±*SD*) were the subjects of this study. They fulfilled the following criteria: no skeletal or dental malocclusion judged by cephalometric criteria according to Ueda, Ishizuka, Miyamoto, Morimoto, and Tanne (1998), no signs and symptoms of temporomandibular disorder, and no previous orthodontic treatment experience. The study protocol was approved by the ethics committee of Tokushima University Hospital (No. 2707) and an informed consent was obtained from each participant.

**Figure 1:**
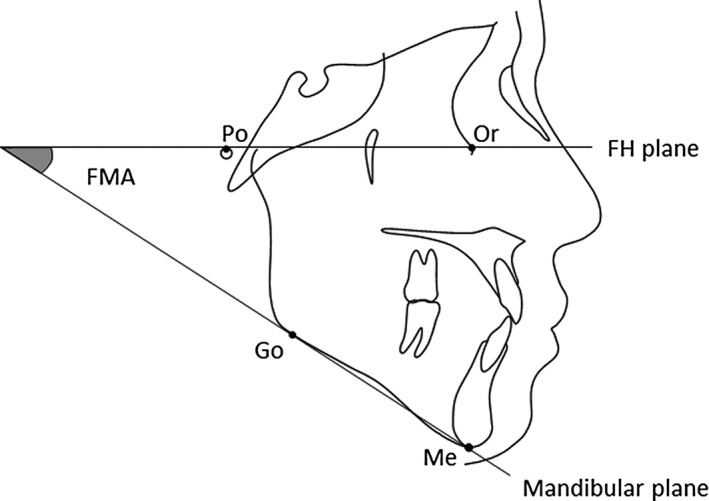
Schematic drawing of the cephalometric analysis. FMA is an angle between mandibular and FH planes

Craniofacial morphology was evaluated on a lateral cephalogram (Figure 1). Each lateral cephalogram was traced on acetate paper by one examiner. Accuracy of the tracing was confirmed by the two orthodontic professionals joining in this study as collaborators. Based on these measurements, the subjects were divided into three groups according to the FMA (angle between mandibular and FH planes); the brachy facial group (subjects with FMA <22°), the mesio facial group (FMA ≥22° but <33°), and the dolicho facial group (FMA >33°).

### 2.2 Gum chewing exercise

Volunteers consented to the gum chewing exercise and reported that they did not regularly chew gum. They were instructed to chew the gum (XYLITOL, OralCare Inc., Tokyo, Japan) for 5 min twice a day for 4 weeks. The MBF was measured at the start (T1) and end of the 4‐week exercise (T2).

### 2.3 Bite force measurements

**Figure 2:**
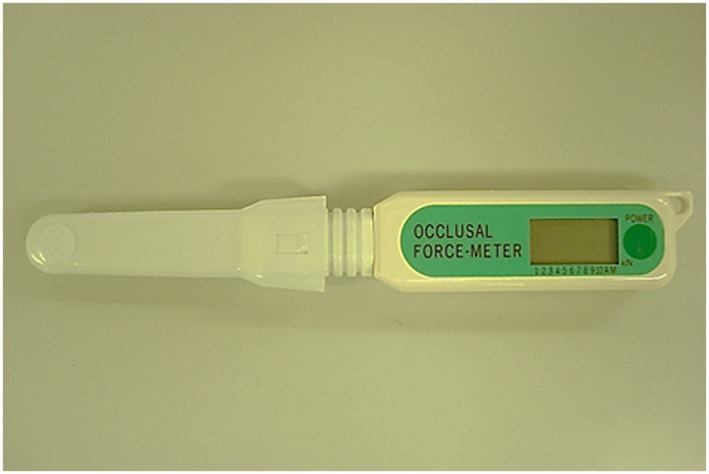
Occlusal Force‐meter GM10 (Nagano Keiki Co. Ltd., Tokyo, Japan)

Bite force was measured unilaterally on both sides in the first molar region using a portable occlusal force gauge (Occlusal Force‐meter GM10, Nagano Keiki Co. Ltd., Tokyo, Japan; Figure 2). The subjects were seated with the Frankfort plane nearly parallel to the floor and instructed to bite on the biting element as hard as possible for 3 s. The force gauge displayed the highest recorded value. Bite force was measured alternately twice on each side with 30‐s interval after each biting. The larger measurement achieved on each side was regarded as representative, and the mean value of bilateral representative measurements was used as the MBF of the individual.

### 2.4 Measurement error study

The bite force and cephalometric variables were measured twice, 1 week apart by one examiner. To test intraobserver reliability, intraclass correlation coefficient was calculated. The intraclass correlation coefficients were 0.9 or more, indicating a small method error.

### 2.5 Statistical analysis

Results of MBF were expressed as mean ± standard error of the mean. Intergroup differences were analyzed by the paired *t* test or analysis of variance. The Bonferroni/Dunn procedure was used as *a post hoc* test when analysis of variance was significant. A *p* value less than .05 was considered statistically significant. All tests were conducted using SPSS 16 software (SPSS Japan).

## 3 RESULTS

Based on measurement of the FMA, seven subjects (two males and five females) were included in the brachy facial group, seven subjects (three males and four females) in the mesio facial group, and five subjects (two males and three females) in the dolicho facial group. Their ages ranged between 22 and 38 years, with a mean of 25.0 ± 1.8, 25.9 ± 4.2, and 25.4 ± 6.3 years (±*SD*) respectively. (Table 1).

**Table 1:** Mean and standard deviations (*SD*) of age and FMA in the three groups

	Number		Age (year)	FMA (°)
	Male	Female		
All subjects	7	12	25.4 ± 4.3	28.0 ± 7.2
Brachy facial	2	5	25.0 ± 1.8	20.3 ± 3.4
Mesio facial	3	4	25.9 ± 4.2	29.7 ± 3.4
Dolicho facial	2	3	25.4 ± 6.3	36.4 ± 1.9

**Figure 3:**
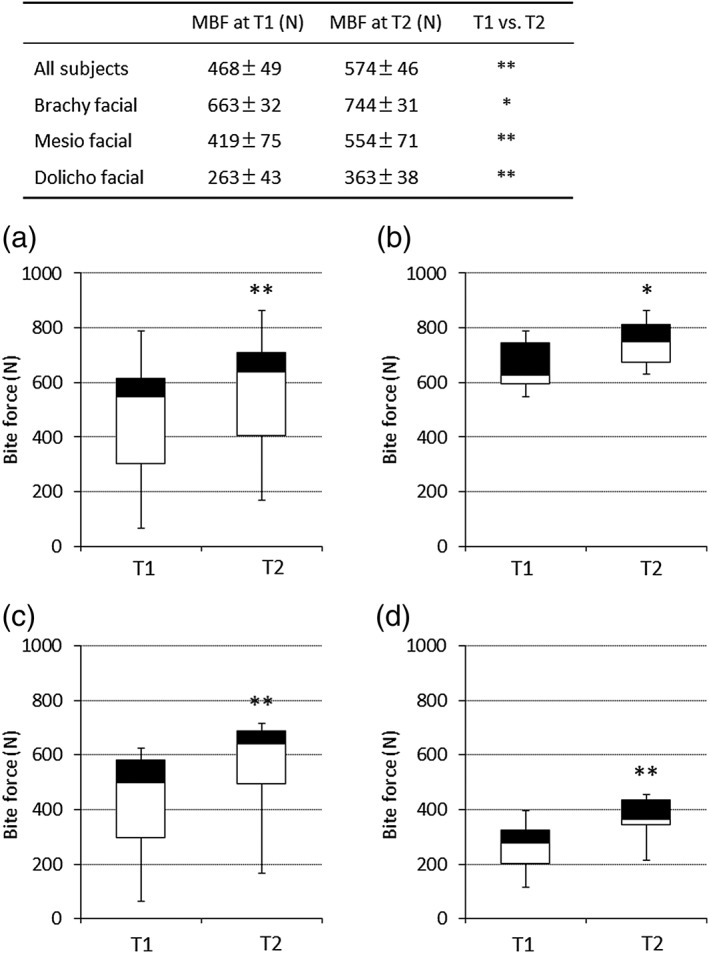
Effects of the gum chewing exercise on maximum bite force (MBF) in subjects of the three facial types. T1 = at start of the chewing exercise. T2 = at end of the chewing exercise. (a) All subjects. (b) Brachy facial group. (c) Mesio facial group. (d) Dolicho facial group. Data are mean ± standard error of the mean. *p < .05, **p < .01 (paired *t* test)

The mean and standard error of the mean of MBF at T1 for the brachy, mesio, and dolicho facial groups were 663 ± 32, 419 ± 75, and 263 ± 43 N, respectively. The mean MBF of the brachy facial group was the largest in three groups and there were significant differences among three groups, mutually (*p* < .01). The MBF from T1 to T2 increased significantly after the gum chewing exercise in all groups. The percent increase in MBF was 22.6% for all the 19 subjects. Among the three groups, the largest increase was noted in the dolicho facial type (37.8%), followed by the brachy facial type (32.2%; Figure 3).

## 4 DISCUSSION

This study investigated the relationship between MBF and facial morphology and effectiveness of the chewing exercise on this parameter. To our knowledge, this is the first study that examines the effectiveness of the chewing exercise on MBF of different facial types.

The gum chewing exercise has been used previously to improve bite force and masticatory function (Kiliaridis et al., 1995; Masumoto et al., 2009; Nakagawa et al., 2017; Ohira et al., 2012; van Bruggen et al., 2015). In this study, the mean MBF value of all subjects increased from 468 to 574 N after 4‐week chewing exercise. This result is in agreement with that of Ohira et al. (2012), who also reported improvement of MBF after 4‐week chewing exercise. However, our results also showed that the magnitude of such increase in MBF was dependent on the facial type, with the highest percentage of increase noted in subjects of the dolicho facial group. Masseter muscle activity (Ueda et al., 1998) and thickness (Satiroğlu, Arun, & Işik, 2005) showed significant negative correlations with vertical craniofacial morphology. The development of the masticatory system is based on functional needs. Especially, jaw muscle function has a considerable effect on craniofacial morphology. We reported previously in soft diet‐fed rats that a decrease in masticatory demand led to masseter muscle atrophy (Kawai et al., 2010) and inferior mandibular development (Hichijo et al., 2014; Hichijo, Tanaka, Kawai, van Ruijven, & Langenbach, 2015). The low muscle performance might be the reason of the effectiveness of chewing exercise in the dolicho facial subjects. These results imply the potential usefulness of gum chewing exercise in improving masticatory performance especially in dolicho facial individuals.

The limitations of this study is that we did not follow‐up the subjects after the end of the exercise to determine the sustainability of the effects. Ohira et al. (2012) reported that the increased MBF of the preschool children by 4‐week gum chewing exercise was maintained for 4 weeks after exercise had finished. However, there has been few studies to investigate the long‐term effect of chewing exercise. Additional limitation was a small number of study sample. In this study, there was a significant difference between MBF of T1and T2, but the study with a small number of sample might lead to Type I error. Therefore, the follow‐up study with sufficient sample number is needed in the future to confirm the sustainability of the effects.

In conclusion, this study demonstrated that gum chewing exercise could be effective in improving masticatory performance, especially in individuals with dolicho facial type.

## CONFLICT OF INTEREST

The authors declare that there are no conflicts of interest with respect to the research and publication of this article.
